# Systematic dissection of genomic features determining the vast diversity of conotoxins

**DOI:** 10.1186/s12864-023-09689-4

**Published:** 2023-10-09

**Authors:** Jian-Wei Zheng, Yang Lu, Yu-Feng Yang, Dan Huang, Da-Wei Li, Xiang Wang, Yang Gao, Wei-Dong Yang, Yuanfang Guan, Hong-Ye Li

**Affiliations:** 1 Key Laboratory of Aquatic Eutrophication and Control of Harmful Algal Blooms of Guangdong Higher Education Institute, College of Life Science and Technology, Jinan University, Guangzhou, 510632 China; 2 College of Food Science and Engineering, Foshan University of Science and Technology, Foshan, 528231 China; 3 Gulou Hospital, Nanjing University, Nanjing, China; 4 Department of Computational Medicine and Bioinformatics, University of Michigan, Ann Arbor, MI USA

**Keywords:** *Conus*, Conotoxin, Gene feature, Transposon element, Introgression

## Abstract

**Background:**

*Conus*, a highly diverse species of venomous predators, has attracted significant attention in neuroscience and new drug development due to their rich collection of neuroactive peptides called conotoxins. Recent advancements in transcriptome, proteome, and genome analyses have facilitated the identification of conotoxins within *Conus*’ venom glands, providing insights into the genetic features and evolutionary patterns of conotoxin genes. However, the underlying mechanism behind the extraordinary hypervariability of conotoxins remains largely unknown.

**Results:**

We analyzed the transcriptomes of 34 *Conus* species, examining various tissues such as the venom duct, venom bulb, and salivary gland, leading to the identification of conotoxin genes. Genetic variation analysis revealed that a subset of these genes (15.78% of the total) in *Conus* species underwent positive selection (Ka/Ks > 1, *p* < 0.01). Additionally, we reassembled and annotated the genome of *C. betulinus*, uncovering 221 conotoxin-encoding genes. These genes primarily consisted of three exons, with a significant portion showing high transcriptional activity in the venom ducts. Importantly, the flanking regions and adjacent introns of conotoxin genes exhibited a higher prevalence of transposon elements, suggesting their potential contribution to the extensive variability observed in conotoxins. Furthermore, we detected genome duplication in *C. betulinus*, which likely contributed to the expansion of conotoxin gene numbers. Interestingly, our study also provided evidence of introgression among *Conus* species, indicating that interspecies hybridization may have played a role in shaping the evolution of diverse conotoxin genes.

**Conclusions:**

This study highlights the impact of adaptive evolution and introgressive hybridization on the genetic diversity of conotoxin genes and the evolution of *Conus*. We also propose a hypothesis suggesting that transposable elements might significantly contribute to the remarkable diversity observed in conotoxins. These findings not only enhance our understanding of peptide genetic diversity but also present a novel approach for peptide bioengineering.

**Supplementary Information:**

The online version contains supplementary material available at 10.1186/s12864-023-09689-4.

## Background

Marine cone snails, belonging to the genus *Conus*, comprise approximately 700 species, and they are typically classified into three main groups: vermivorous, molluscivorous, and piscivorous [1]. These carnivorous predators utilize conotoxins, found in their milked venom, for prey hunting. Conotoxins are intricate combinations of small-molecule active polypeptides known as conopeptides, which generally consist of 30 to 200 amino acid residues and can adopt diverse disulfide structures [2, 3].

The precursor of conopeptides typically consists of three domains: an N-terminal signal peptide, a propeptide, and a mature peptide located near the C-terminal. Among these domains, the signal peptide exhibits high conservation within the gene superfamily [4]. In contrast, the mature peptides display significant variability among conopeptides. Analysis of mature peptides reveals an accelerated rate of nucleotide substitution and a predominance of nonsynonymous substitutions, suggesting that the targeted mutators in the mature peptide region and diversifying selection may account for the hypervariability observed in conopeptides [5]. Similarly, *C. bullatus* demonstrates high structural diversity and a high single-nucleotide polymorphism (SNP) rate in conopeptides, supporting the hypothesis of diversifying selection in conopeptides [6].

Furthermore, targeted sequencing of venom genes from 32 *Conus* species has revealed a wide range of conotoxin gene copies, varying from 120 to 859. Notably, exons encoding the mature toxin region exhibit higher divergence, indicating positive selection acting on conotoxin genes [7]. However, the precise factors influencing the genetic hypervariability of conotoxin genes remain unclear.

The genomes of four *Conus* species have been released, including *C. tribblei* [8], *C. consors* [9], *C. betulinus* [10], and *C. ventricosus* [11]. However, the genomes of *C. tribblei* and *C. consors* suffer from severe fragmentation, which limits further analysis. On the other hand, the high-quality genome of *C. betulinus* provides valuable insights into the fundamental genetic principles governing conopeptides. Notably, it reveals a primary genetic relationship known as the “central dogma” of conopeptides, where the ratio of genes to transcripts to proteins to conopeptides is approximately 1:1:1:10. This observation suggests that post-translational modifications, such as alternative cleavage sites, highly variable N- and C-terminal truncations, and post-translational modifications, may play a significant role in generating the extensive diversity of conopeptides derived from a limited set of conotoxin genes [10, 12]. These findings significantly advance our understanding of conopeptide diversity at the translational and post-translational modification levels.

However, the genetic evolution of conotoxin genes remains a subject of ongoing investigation. Moreover, the lack of available genome annotation for the published *C. betulinus* genome hinders its comprehensive utilization in further analysis. Additionally, noncoding regions of the genome have been shown to contribute to gene diversity. Introns, which are prevalent in Metazoan genomes, facilitate frequent alternative splicing and promote the diversification of gene families through exon recombination [13]. Similarly, transposon elements (TEs) play crucial roles in genome evolution and function, including genome mutations, rearrangements, and the generation of new genes [14]. The chromosome-level genome of *C. ventricosus* suggests that conotoxin genes are located within repetitive regions, and a whole-genome duplication event has been identified [11]. These findings indicate a potential association between genome features and the diversity of conotoxin genes. In summary, while extensive research has been conducted on conotoxins, the molecular mechanisms underlying the genetic diversity of conotoxin genes remain largely unresolved. Further investigation is warranted to explore the intricate relationships between genome features and the hypervariability observed in conotoxin genes.

In this study, we conducted transcriptome assembly for 34 *Conus* species to analyze the genetic evolution of conotoxin genes. However, due to the unavailability of publicly accessible genome annotation for the published *C. betulinus* genome, which limits comprehensive analysis of its genomic structure, we performed additional reassembly of the complete genome of *C. betulinus* using publicly available genome sequencing datasets from Peng et al. [10]. Through this process, we annotated repetitive elements and protein-coding genes, enabling us to investigate whole genome duplication events, structural characteristics, expression patterns, and alternative splicing processes of conotoxin genes in *C. betulinus*. Moreover, we explored the presence of transposable elements in the flanking regions of conotoxin genes and in the introns adjacent to the highly variable mature-peptide coding sequences. Additionally, we assessed the occurrence of introgressive hybridization of conotoxin genes among various *Conus* species, utilizing publicly available conotoxin gene targeted sequencing datasets from Phuong et al. [7].

## Results

### Transcriptome assembly and evaluation of 34 *Conus* species

RNA sequencing datasets of 34 *Conus* species were retrieved from NCBI (Table S1), and transcripts of each species were assembled. The number of unigenes among different species varied greatly, as shown in Table 1, ranging from 20,062 to 235,341. Similarly, N50 and completeness of transcripts in each species were also diverse in the range from 290 bp to 1,213 bp in N50 and 21.6–95.7% in completeness (percentage of complete and fragmented BUSCOs), respectively. In addition, there were 21 species of *Conus* for which the “complete and fragmented BUSCOs” were higher than 50%.

**Table 1:** Summary of transcriptome assembly of 34 *Conus* species

Species	No. of Unigenes	N50 (bp)	BUSCO (%) ^a^	Species	No. of Unigenes	N50 (bp)	BUSCO (%) ^a^
*C. abbreviatus*	64,205	469	45.5	* C. magus*	95,345	917	77.4
* C. arenatus*	19,861	584	29.7	* C. maioensis*	100,467	890	72.9
* C. aristophanes*	73,258	489	53.3	* C. marmoreus*	89,231	818	78.4
* C. bayani*	135,227	549	78.0	* C. miliaris*	123,758	695	80.2
* C. betulinus*	235,341	677	95.4	* C. mordeirae*	83,999	628	67.6
* C. chaldaeus*	123,872	557	64.9	* C. purpurascens*	181,074	1,128	95.7
* C. consors*	179,498	1,213	94.2	* C. quercinus*	25,760	617	36.1
* C. coronatus*	31,974	511	41.5	* C. rattus*	23,807	616	40.7
* C. ebraeus*	46,323	583	49.7	* C. regonae*	77,176	587	62.4
* C. episcopatus*	71,367	629	21.6	* C. sp.* f AW-2021	91,192	290	34.2
* C. ermineus*	104,136	1,029	78.5	* C. sponsalis*	20,062	582	27.6
* C. gloriamaris*	178,627	512	69.3	* C. striatus*	108,738	854	86.4
* C. imperialis*	116,677	707	80.6	* C. terebra*	50,768	622	49.4
* C. judaeus*	80,425	324	46.5	* C. textile*	66,371	426	47.1
* C. lenavati*	203,012	542	84.2	* C. tribblei*	182,538	776	87.9
* C. litteratus*	88,210	428	58.0	* C. ventricosus*	119,838	1,028	79.7
* C. lividus*	25,887	575	38.9	* C. virgo*	106,353	892	87.3

**a**: Percentage of complete and fragmented BUSCOs.

### Identification and genetic variation of conotoxins in *Conus*

**Fig. 1:**
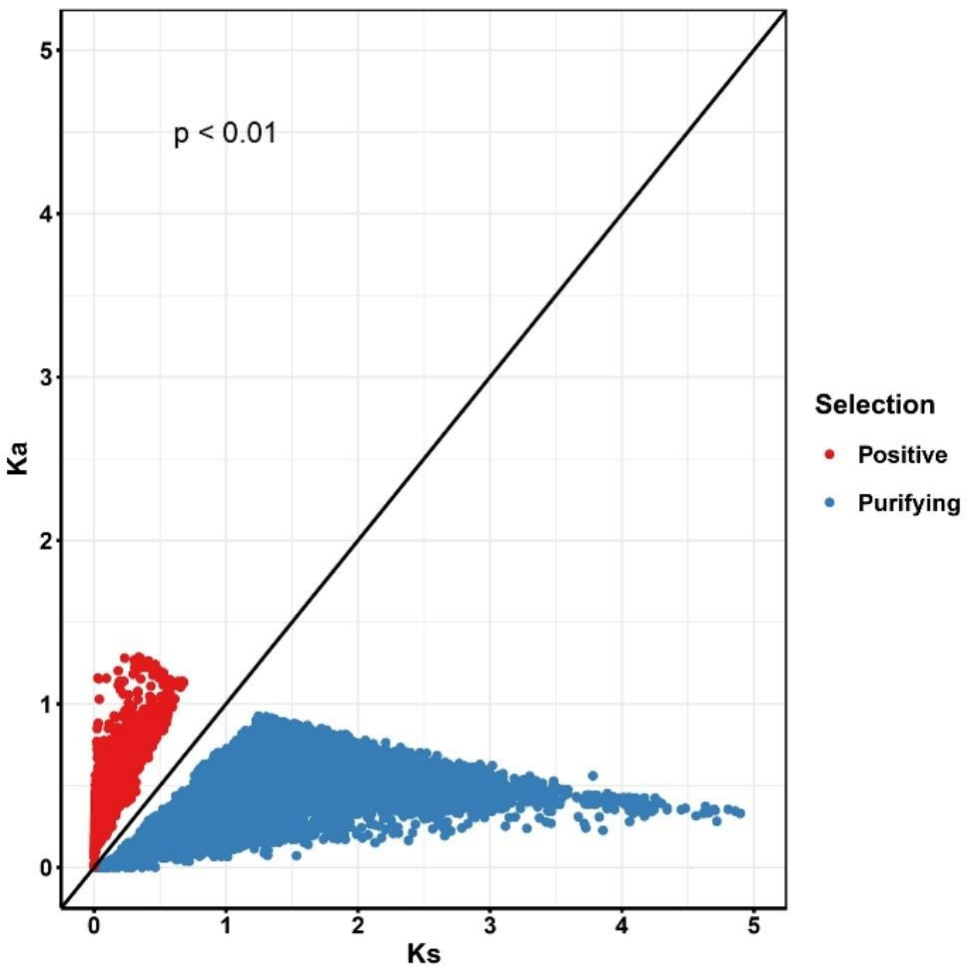
Ka/Ks calculation of conotoxin genes orthogroups in 34 *Conus* species

Conotoxins were predicted in each species of *Conus*. A total of 4,111 conotoxins were identified in 34 *Conus* species transcriptomes from published data (Table S1), and the number of identified conotoxins in each species varied widely, ranging from 34 to 282 (Table 2). Subsequently, homology clustering was performed on the total conotoxin genes, and 91 orthogroups were archived. Among these orthogroups, there were 22 orthogroups that each contained more than 80% of the species represented, including 4 orthogroups that contained all species represented. Genetic variation analysis of conotoxins within orthogroups showed that most conotoxins were under purifying selection (Ka/Ks < 1) (Fig. 1). However, positive selection (Ka/Ks > 1, total of 15.78%) and significant genetic variation were also observed in conotoxins.

**Table 2:** Identified conotoxins in 34 *Conus* species

Species	No. of conotoxins	Species	No. of conotoxins
*C. abbreviatus*	253	*C. magus*	135
*C. arenatus*	129	*C. maioensis*	154
*C. aristophanes*	282	*C. marmoreus*	188
*C. bayani*	119	*C. miliaris*	127
*C. betulinus*	65	*C. mordeirae*	153
*C. chaldaeus*	83	*C. purpurascens*	51
*C. consors*	57	*C. quercinus*	73
*C. coronatus*	206	*C. rattus*	94
*C. ebraeus*	111	*C. regonae*	178
*C. episcopatus*	34	*C. sp.* f AW-2021	123
* C. ermineus*	58	*C. sponsalis*	159
*C. gloriamaris*	89	*C. striatus*	107
*C. imperialis*	102	*C. terebra*	81
*C. judaeus*	124	*C. textile*	99
*C. lenavati*	81	*C. tribblei*	61
*C. litteratus*	145	*C. ventricosus*	86
*C. lividus*	105	*C. virgo*	199

**Positive**: positive selection (Ka/Ks > 1). **Purifying**: purifying selection (Ka/Ks < 1).

### Genome reassembly and annotation of *C. betulinus*

A total of 239.7 Gb of raw reads generated by PacBio Sequel were used to assemble the genome of *C. betulinus* and polished with cleaned Illumina sequencing reads, resulting in an assembly of 51,913 scaffolds with a total length of 2.67 Gb. The assembly genome results showed that the maximum and average scaffold lengths were 2.66 Mb and 51.5 kb, respectively, with an N50 length of 127 kb (Table 3). Meanwhile, in addition to simple repeats, LINEs (long interspersed nuclear elements, 9.71% of the genome) had the highest proportion and were the main types of transposon elements (Table S2). LTRs (long terminal repeats, 6.88% of the genome) were also abundant in the genome of *C. betulinus.* It has been reported that both LINEs and LTRs are retrotransposons [14].

**Table 3:** Summary of the reassembly genome of *C. betulinus*

Genome evaluation	This study (reassembled)	Peng et al. [10]
Scaffold number	51,913	41,426
Total bases (bp)	2,673,840,836	3,430,828,710
Max sequence length (bp)	2,659,028	2,850,889
Average sequence length (bp)	51,506.19	82,815.21
Median sequence length (bp)	20,689	31,036
N50 (bp)	127,191	232,607
Ns (%)	1.53	0.87
Protein-coding gene number	24,308	22,698
Complete BUSCO score (%)	83.8	89.8

Combined with *de novo*, homology, and RNA-seq methods, we finally predicted 24,308 protein-coding genes in *C. betulinus*. Overall, the transcription of 97.7% of the protein-coding genes was supported by the transcriptomes of multiple specimens. Moreover, BUSCO with the metazoa_odb10 database was employed to evaluate the completeness of predicted genes. It showed that the predicted genes contained 688 (72.1%) single-copy and 112 (11.7%) duplicated complete genes, as well as 49 (5.1%) fragmented genes. Compared with the published genome of *C. betulinus* [10], which contained 763 (78.0%) single-copy and 115 (11.8%) duplicated complete genes and 31 (3.17%) fragmented BUSCOs, genome completeness was similarly high in the present study.

**Fig. 2:**
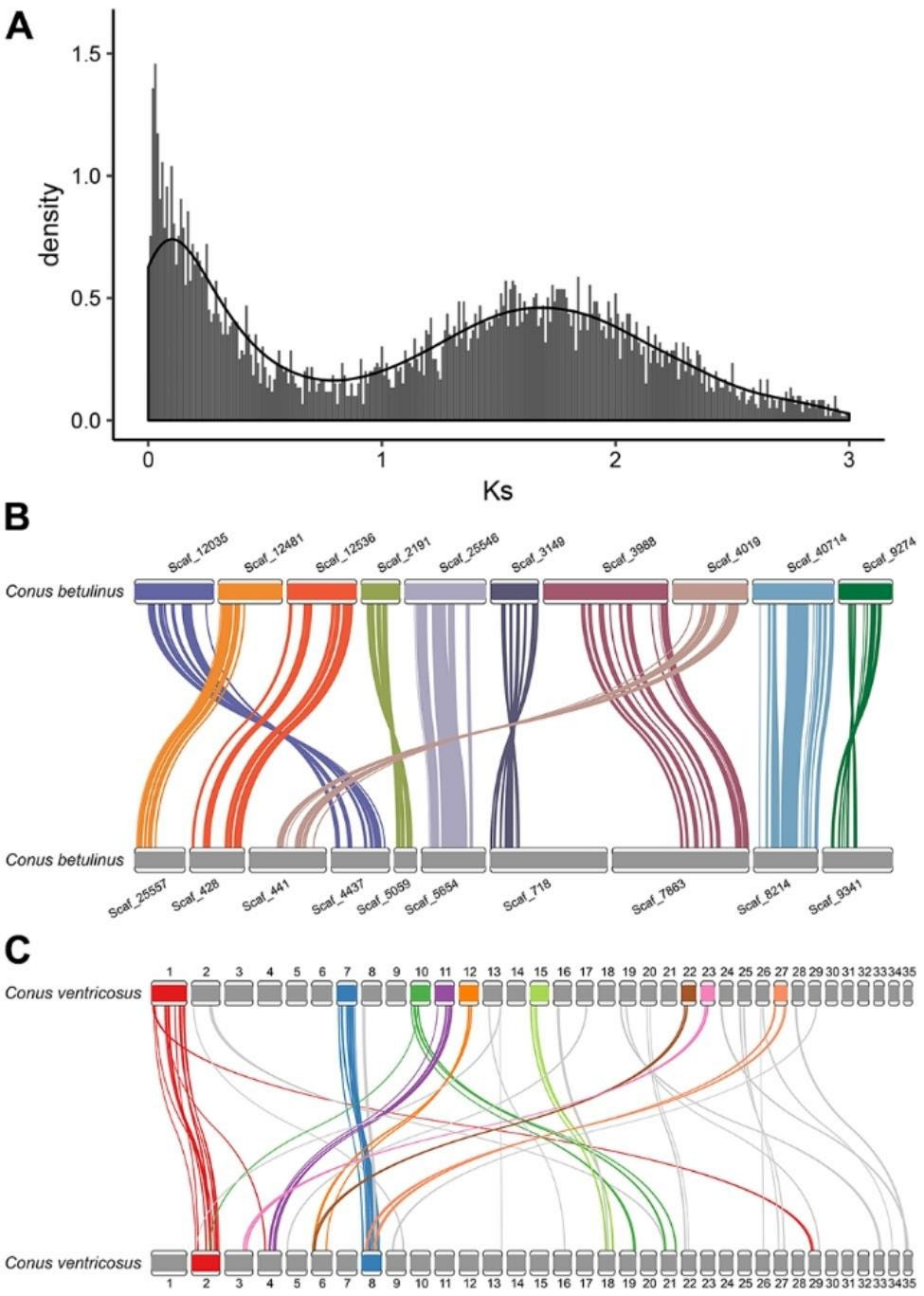
Whole genome duplication (WGD) and conserved homologous gene blocks analysis of *C. betulinus* and *C. ventricosus*

Subsequently, as shown in Fig. 2A, the distribution of synonymous substitution rate (Ks) between paralog pairs in *C. betulinus* was calculated. And a second Ks peak was observed, suggesting similar divergence between paralogs after whole genome duplication (WGD). Furthermore, significantly conserved homologous gene blocks were identified among the scaffolds of the *C. betulinus* genome (Fig. 2B). Similarly, conserved homologous gene blocks among the pseudo-chromosomes of the *C. ventricosus* genome were also observed (Fig. 2C), for instance, between pseudo-chromosomes 1 and 2, and between pseudo-chromosomes 7 and 8.

**(A)** Distribution of synonymous substitution rate (Ks) between paralog pairs in *C. betulinus*. The presence of second Ks peak suggests the similar divergence between paralogs after WGD. **(B)** Conserved homologous gene blocks between scaffolds in *C. betulinus* derived from ortholog proteins. **C.** Conserved homologous gene blocks between chromosome-level scaffolds in *C. ventricosus* derived from ortholog proteins.

### Expression characteristics of conotoxin genes in *C. betulinus*

**Fig. 3:**
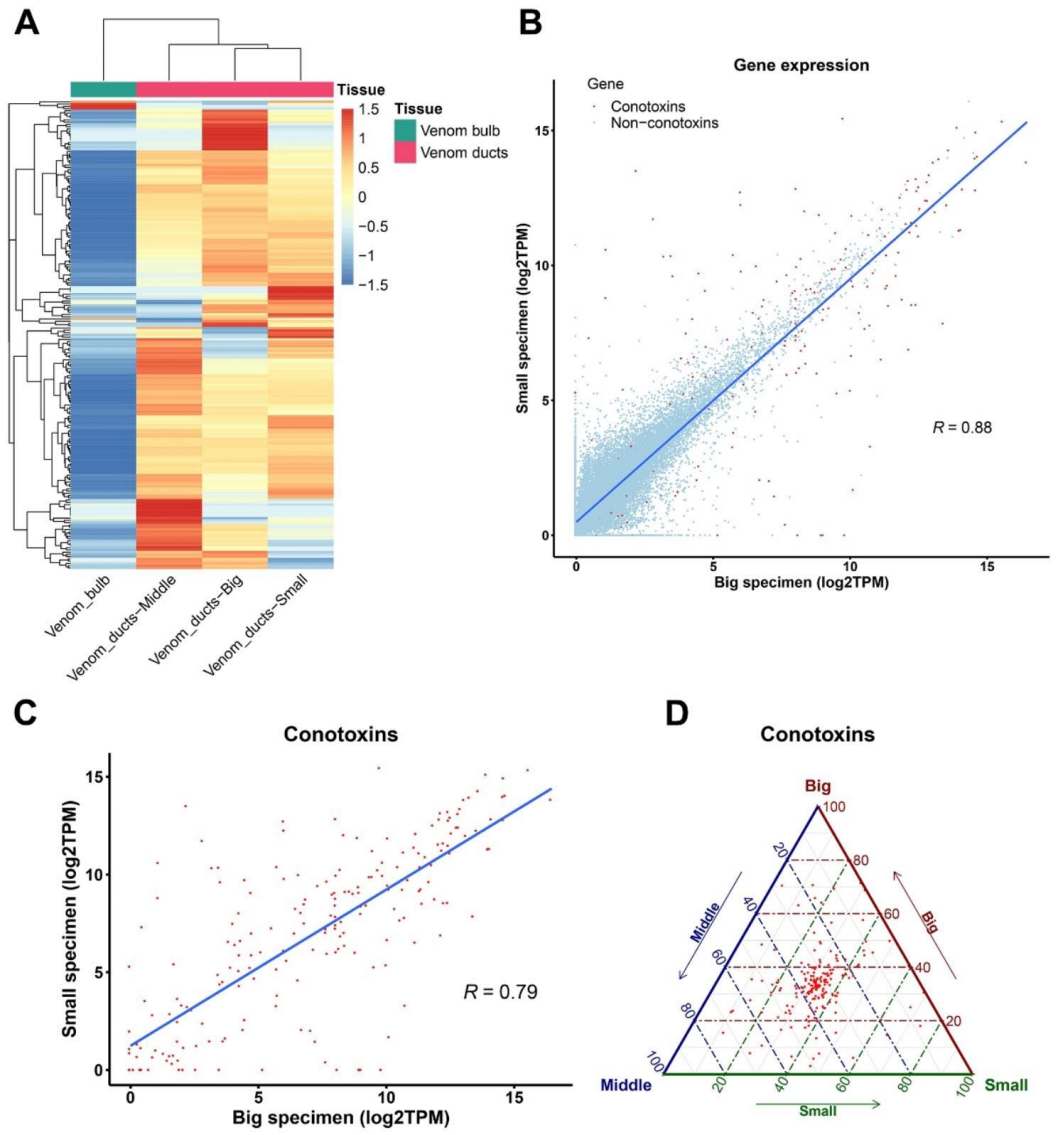
Expression characteristics of conotoxin genes in different tissues and specimens of *C. betulinus. ***A.** Expression (TPM, transcripts per kilobase million) of conotoxin genes in different tissues (venom bulb and venom ducts) of *C. betulinus*. TPM was normalized by z-score. **B.** Expression of conotoxin and non-conotoxin genes in venom ducts between different body length individuals (small and big) of *C. betulinus*. Red color: conotoxin genes, blue color: non-conotoxin genes. **C - D.** Expression of conotoxin genes in venom ducts between different body length individuals (small, middle and big) of *C. betulinus*

In the present study, 221 conotoxin genes were identified in the reassembled *C. betulinus* genome. Whereas 133 conotoxin genes are identified in the published genome [10]. Homology clustering results on these two gene sets showed that 117 and 43 conotoxin genes were uniquely identified in the reassembled and published genomes, respectively (Table S3). The identified conotoxin genes in the present study were classified into 12 known superfamilies, and the M- and O- superfamilies were the most abundant. Meanwhile, 17 cysteine frameworks were also classified. However, 142 conotoxin genes were unclassified into the known superfamilies. The expression level (TPM) of those 221 conotoxin genes in the venom bulbs and venom ducts from different body lengths of *C. betulinus*, namely small, middle, and big, was calculated and used to profile the expression patterns of conotoxin genes. It showed that the expression level of conotoxin genes in venom ducts was dramatically higher than that in the venom bulb (Fig. 3A), and in venom ducts, 169 out of 221 (76.47%) conotoxin genes had an average TMP higher than 10. Meanwhile, although conotoxin genes were highly expressed in all venom duct tissues, the expression characteristics of conotoxin genes in the venom ducts of individuals with different body lengths were significantly diverse. As shown in Fig. 3B, the expression level (TPM) of most genes in the venom ducts of small and big individuals was relatively consistent, especially the non-conotoxin genes. However, the expression level of conotoxin genes in the venom ducts between individuals with different body lengths, including small, middle, and big, was significantly diverse (Fig. 3C and D).

Additionally, alternative splicing has been observed in the transcription process of conotoxin genes among individuals with different body lengths, which may increase the diversity of conotoxins in *C. betulinus*. However, the frequency of alternative splicing of conotoxin genes between different individuals was limited, with only 24 conotoxin genes detected.

### Structure features of conotoxin genes in *C. betulinus*

**Fig. 4:**
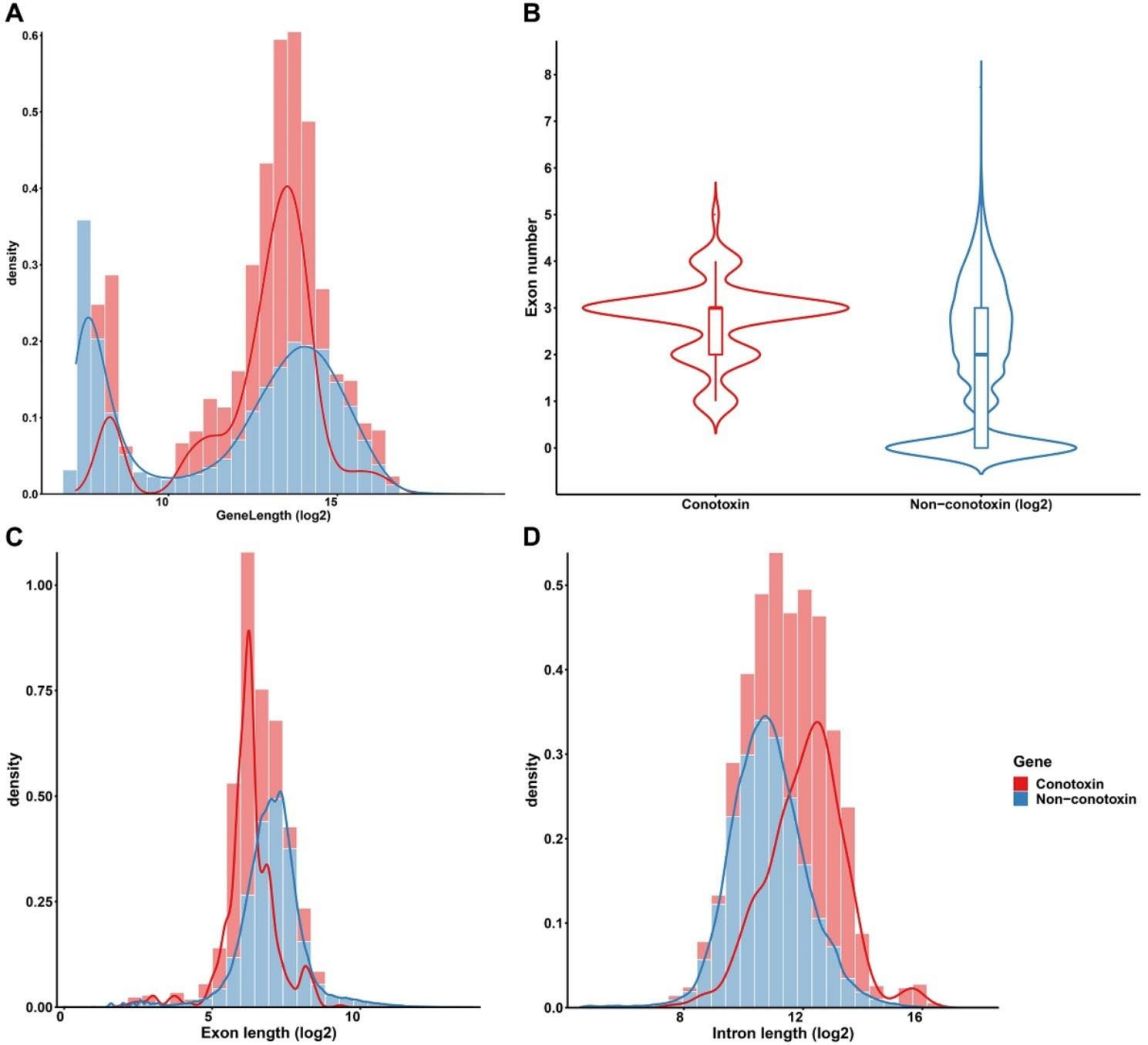
Structure features of protein-coding genes in *C. betulinus. ***A.** Distribution of protein-coding genes length. **B.** Statistics of exon numbers in protein-coding genes. **C.** Distribution of exon length. **D.** Distribution of intron length. **Red color**: conotoxin genes. **Blue color**: non-conotoxin genes

The distribution of the length of protein-coding genes in *C. betulinus* showed that the length of conotoxin and non-conotoxin genes was relatively similar; the average length of conotoxin and non-conotoxin genes was 12,014 bp and 14,063 bp, respectively (Fig. 4A). In addition, the statistical results of the exon number of protein-coding genes showed that the conotoxin genes were mainly composed of three exons, while the exon number of non-conotoxin genes showed greater fluctuation (Fig. 4B). Furthermore, the statistical results of the exon length of protein-coding genes in *C. betulinus* showed that the exon length of non-conotoxin genes was approximately double that of conotoxin genes, with the average exon length of conotoxin and non-conotoxin genes being 92 and 177 bp, respectively (Fig. 4C). It’s worth noting that the intron length of conotoxin genes in *C. betulinus* was significantly longer than that of non-conotoxin genes (Fig. 4D); however, the effects of introns, such as providing mutational hotspots [15] or affecting gene size or structure expansion [16], on the diversity of conotoxin genes remained largely unknown.

**Fig. 5:**
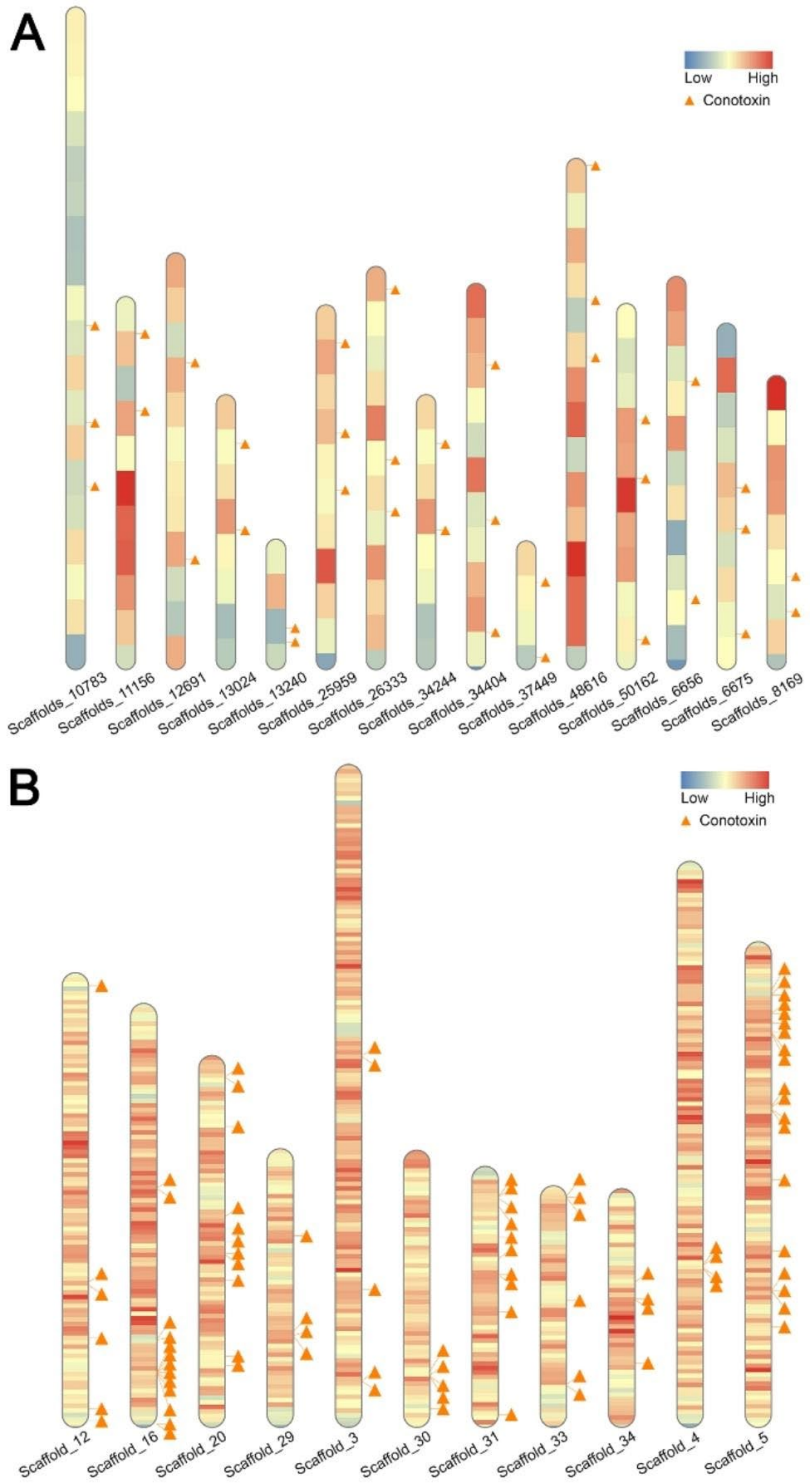
Distribution of conotoxin genes and density of transposable elements (TEs) in parts of the scaffolds and chromosomes of *C. betulinus* and *C. ventricosus. ***A.** Distribution of parts of conotoxin genes and density of TEs in some of *C. betulinus* scaffolds. **B.** Distribution of parts of conotoxin genes and density of TEs in some of *C. ventricosus* chromosomes. **Triangle**: Conotoxin genes located in genome. **Heatmap**: Density of TEs in genome from high (red) to low (blue)

In the present study, gene family expansion and contraction analysis of *C. betulinus* showed that two reverse transcriptases and a transposable element-derived protein had expanded rapidly. TE abundance in the genome of *C. betulinus* was counted with a sliding window of 20 kb. Combined with the distribution of conotoxin genes in the *C. betulinus* genome, we proposed that there may be a high density of TEs around conotoxin genes (Fig. 5A). Considering that the genome of *C. betulinus* assembled in the present study was still fragmented and might affect the statistical results, the chromosome-level genome of *C. ventricosus* published recently [11] was also included in the analysis. It showed that conotoxin genes in *C. ventricosus* tended to be distributed in regions with high TE density (Fig. 5B), suggesting that TEs may be related to the genetic diversity of conotoxin genes.

**Fig. 6:**
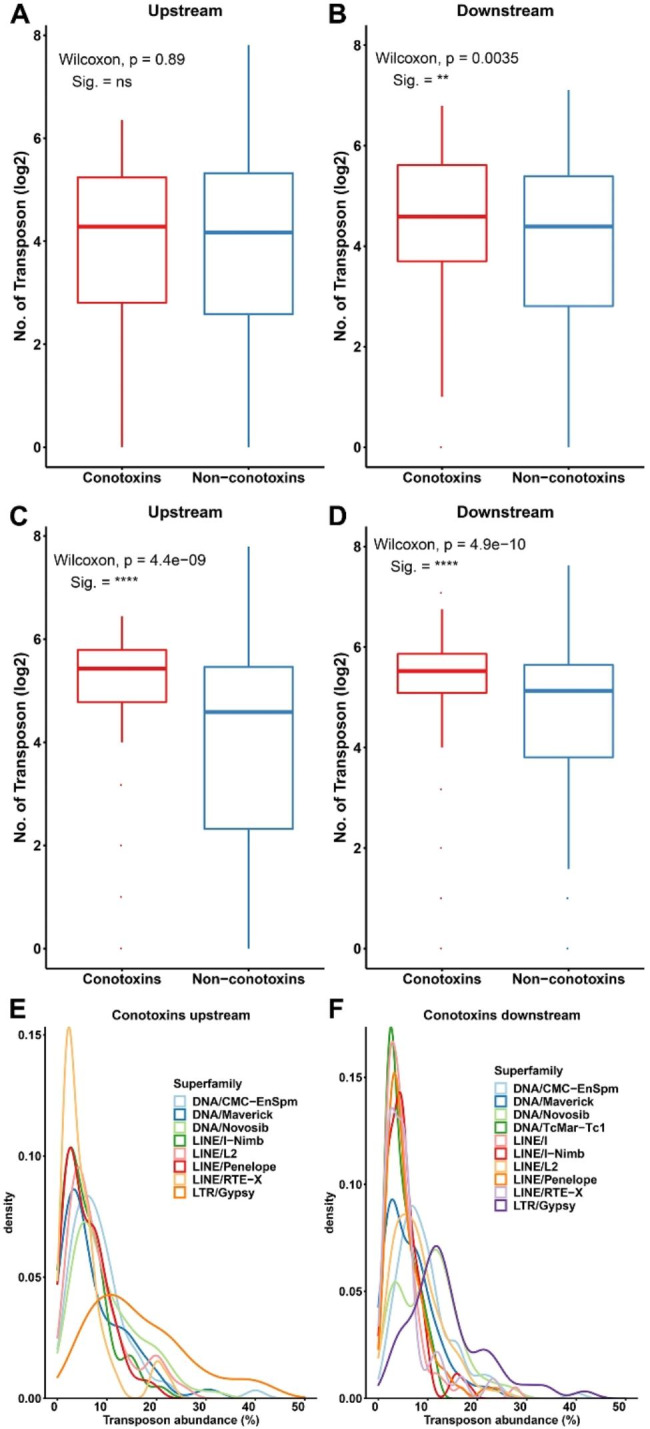
Content of TEs in flanking regions (100 kb) of protein-coding genes in *C. betulinus* and *C. ventricosus. ***A - B.** Content of TEs in the upstream and downstream flanking regions of protein-coding genes in *C. betulinus*. **C - D.** Content of TEs in the upstream and downstream flanking regions of protein-coding genes in *C. ventricosus*. **E - F.** Abundance of different type of TEs in the upstream and downstream flanking regions of conotoxin genes in *C. ventricosus*. Significant differences were performed by Wilcoxon method, and indicated at *p* < 0.01 (**) or *p* < 1e-5 (****)

Subsequently, the content of TEs in the upstream and downstream flanking regions (100 kb) of protein-coding genes in the *C. betulinus* genome was analyzed. As shown in Fig. 6A, there was no significant difference in the content of TEs in the upstream flanking regions between conotoxin and non-conotoxin genes in *C. betulinus*. In contrast, the content of TEs in the downstream flanking regions of conotoxin genes was significantly higher than that in non-conotoxin genes (Fig. 6B, Table S4). Considering that the genome of *C. betulinus* assembled in the present study was still fragmented, the analysis was also carried out on the chromosome-level genome of *C. ventricosus*. It showed that the content of TEs in both the upstream and downstream flanking regions of conotoxin genes was significantly higher than that in non-conotoxin genes in *C. ventricosus* (Fig. 6C and D, Table S5). Moreover, type I retrotransposons (LINE and LTR) and type II DNA transposons are the main TEs in the flanking regions (both upstream and downstream) of conotoxin genes (Fig. 6E F, Table S5). Furthermore, the downstream flanking region of the conotoxin gene had more TEs than the upstream region. In addition, the Gypsy superfamily of LTR retrotransposons has a higher proportion in both upstream and downstream flanking regions of protein-coding genes, ranging from 8.13 to 10.43% in *C. betulinus* and 14.83–16.70% in *C. ventricosus* (Table S4 and Table S5).

**Fig. 7:**
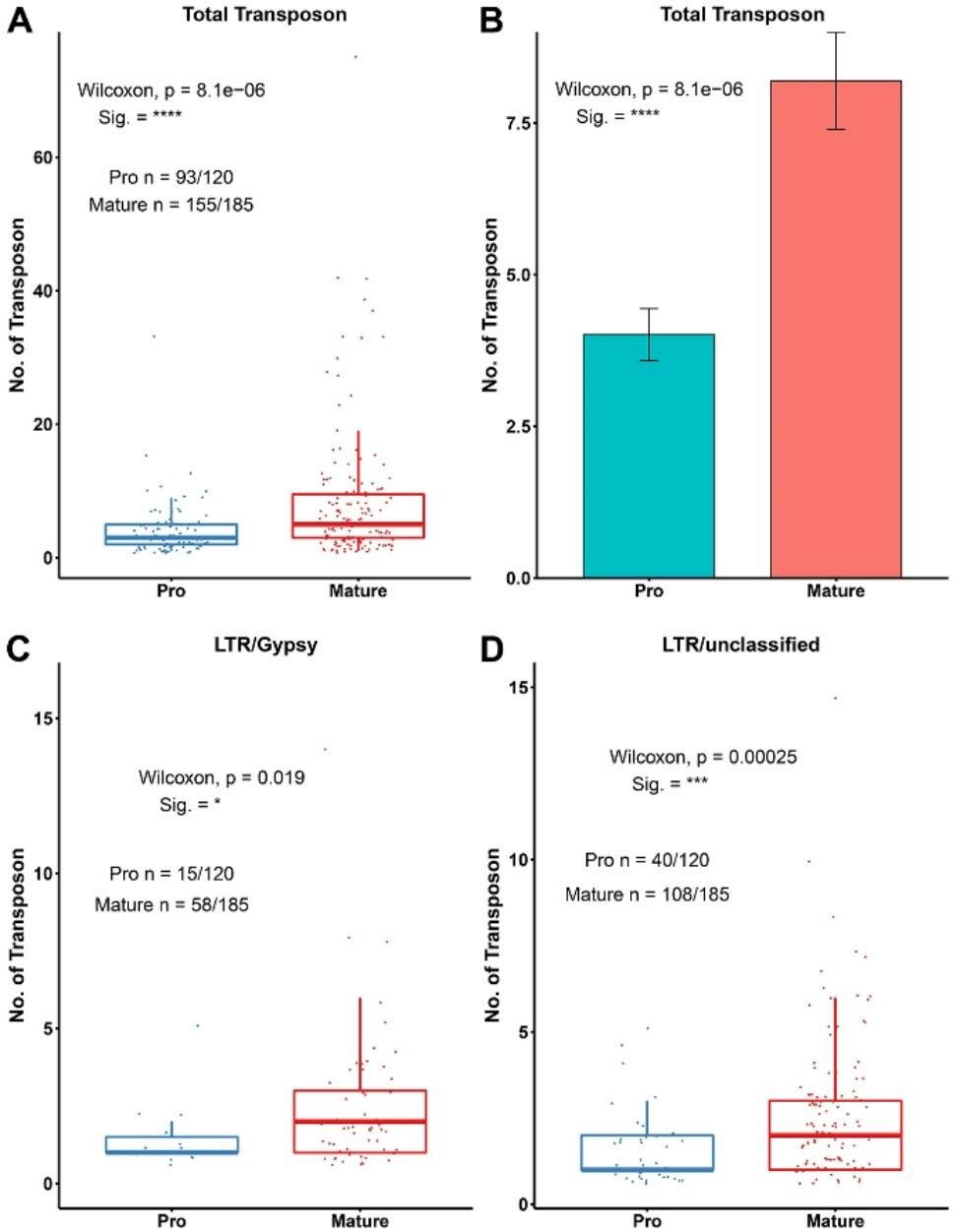
Content of TEs in introns of conotoxin genes in *C. betulinus. ***A - B.** Content of TEs in introns that adjacent to the conotoxin pro- and mature peptide. **C - D.** Content of LTRs in introns that adjacent to the conotoxin pro- and mature peptide. **Pro**: pro-peptide of conopeptides. **Mature**: mature peptide of conopeptide. Significant differences were performed using Wilcoxon method, and indicated at *p* < 0.05 (*), *p* < 0.001 (***) or *p* < 1e-5 (****)

As shown in Fig. 4D, conotoxin genes have longer intron structures compared to those of non-conotoxin genes in *C. betulinus*. The content of TEs in introns adjacent to the highly variable mature peptide of conotoxins was significantly higher than that of introns adjacent to the conserved pro-peptide (Fig. 7A and B, Table S6). Like the main types of TEs in the flanking regions (upstream and downstream) of conotoxin genes, the content of the Gypsy and unclassified families that belong to LTRs in introns adjacent to the mature peptide was also markedly higher than that of introns adjacent to the pro-peptide (Fig. 7C and D, Table S6). However, there was no significant difference between mature peptide and pro-peptide for the retrotransposon of LINEs (data not shown).

### Introgressive hybridization of conotoxin genes in *Conus*

**Fig. 8:**
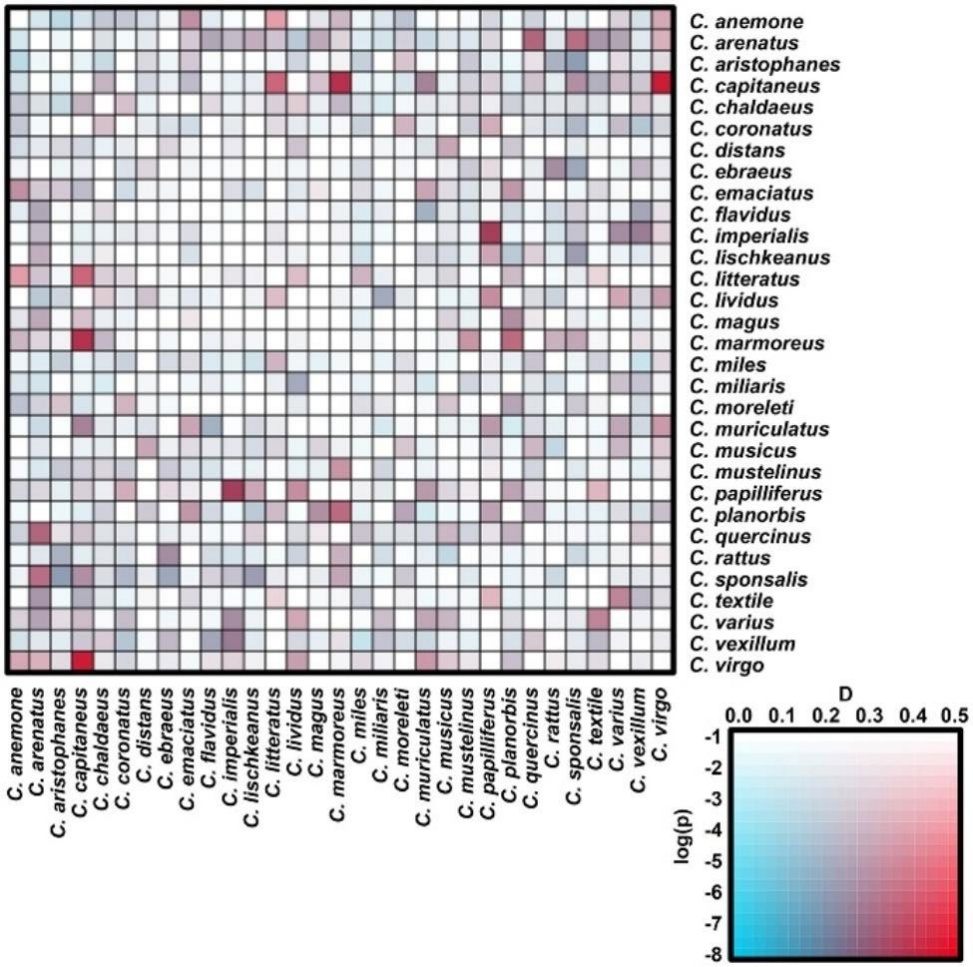
Paterson’s *D* (ABBA-BABA) statistic test of introgression of conotoxin genes in *Conus. ***Legend heatmap**: *D*-statistic value in abscissa and *p* value that transformed by logarithm in ordinate. Redder colors in grid indicate higher introgression level

Introgression among conotoxin genes was detected based on the exon capture targeting sequencing of conotoxin gene loci from 32 *Conus* species [7]. The introgression signals detected by D-statistic are shown in Fig. 8. Most of the *Conus* species pairs showed significant introgression signals. For instance, strong evidence of introgression was detected in *C. capitaneus* and *C. virgo* (*D* = 0.4403, *p*-value = 1.55e-10), *C. capitaneus* and *C. marmoreus* (*D* = 0.3868, *p*-value = 5.50e-11), *C. imperialis* and *C. papilliferus* (*D* = 0.3510, *p*-value = 2.53e-09), and *C. arenatus* and *C. quercinus* (*D* = 0.3396, *p*-value = 1.91e-07). These findings strongly suggested that there was significant gene flow of conotoxin genes between *Conus* species.

## Discussion

In this study, we conducted transcriptome assembly for 34 *Conus* species. However, some species exhibited lower completeness of BUSCOs, which could be attributed to variations in sequencing depth coverage or the tissue types used for sequencing (Table S1). Additionally, the detection of conotoxins varied significantly among the different *Conus* species (Table 2), consistent with previous findings [17–19]. In *C. quercinus*, significant differences in the classes of conotoxin gene superfamilies between the venom duct, venom bulb, and salivary gland were observed, and the transcript activity of conotoxins was lower in both the venom bulb and salivary gland, suggesting that the venom duct is the primary site of conotoxin production [20]. Moreover, significant variations in conotoxins were identified among different individuals of *C. magus*, highlighting the highly diverse nature of conotoxins [21]. In the present study, it also showed that the expression characteristics of conotoxin genes in the venom ducts of *C. betulinus* with different body lengths were significantly diverse, suggesting that the different transcriptional expression or regulation patterns of conotoxin genes during the developmental phases may be one of the factors causing the diversification of conotoxins. Furthermore, we assessed Ka (nonsynonymous nucleotide substitutions) and Ks (synonymous nucleotide substitutions) values for conotoxin genes across the 34 *Conus* species. These parameters play a crucial role in molecular evolutionary analysis, with Ka/Ks > 1, Ka/Ks = 1, and Ka/Ks < 1 generally indicating positive selection, neutral mutation, and purifying selection, respectively [22]. In our study, we found that certain conotoxin genes from the 34 *Conus* species exhibited Ka/Ks values > 1 (*p* < 0.01, Fig. 1), indicating positive selection acting on these genes. This finding aligns with the hypervariability observed in conopeptides [5]. Positive selection facilitates the spread of advantageous mutations, while purifying selection prevents the propagation of detrimental mutations [23]. Therefore, positive selection may contribute to the genetic diversity of conotoxin genes. Likewise, the exons coding the mature peptide of conotoxins exhibited approximately three times higher divergence than their flanking non-coding regions [7].

Conotoxin genes in *Conus* typically consist of 1–6 exons [7]. Our findings revealed that conotoxin genes in *C. betulinus* exhibited a range of exon numbers from 1 to 5, with a predominant composition of 3 exons, similar to *C. ventricosus* [11]. However, it is important to note that these three exons may not align precisely with the three structural domains of conopeptides, namely the signal peptide, pro-peptide, and mature peptide [11]. Interestingly, we observed a longer intron structure in conotoxin genes of *C. betulinus* compared to non-conotoxin genes. It has been observed that Metazoa genomes are enriched with introns, which can provide additional binding sites for transcriptional regulatory elements and facilitate gene diversification through exon recombination [13]. In fungal mitochondria, self-splicing introns have been implicated in increasing the genetic diversity of exons flanking them, suggesting that intron mobility directly influences host gene diversity [15]. Moreover, genes expressed abundantly in the nervous system often exhibit intron and gene size expansion, implying that the unique attributes of neurons may facilitate the evolution of neuronal genes [16]. Consequently, it is worth investigating whether introns in conotoxin genes influence the diversity of conotoxins.

Our results indicated a rapid expansion of two reverse transcriptases and one transposable element-derived protein in *C. betulinus*. Reverse transcriptase is a key enzymatic domain found in all autonomous retrotransposons, as it catalyzes the process of reverse transcription effectively [24]. Class I retrotransposons, one of the major classes of transposable elements (TEs), rely on the activity of reverse transcriptases and integrases [25]. Interestingly, in concurrence with the expansion of TE-related gene families, TEs were found to be highly prevalent in introns adjacent to the hypervariable mature peptide of conotoxins. This suggests that TE hotspots in these specific introns may contribute to the high variability observed in the mature peptide of conotoxins. Similarly, conotoxin genes in *C. ventricosus* are typically found in regions that harbor Class I retrotransposons (Gypsy, Penelope, etc.) and Class II DNA transposons (Tc1-Mariner, etc.) [11]. Consistent with previous studies, our findings indicated that the content of TEs in the flanking regions (upstream and downstream) of conotoxin genes in both *C. betulinus* and *C. ventricosus* was significantly higher compared to non-conotoxin genes (Table S4 and Table S5). Notably, the proportion of the Gypsy superfamily in the flanking regions (combined with upstream and downstream) of conotoxin genes was particularly prominent (9.45% of major TEs in *C. betulinus* and of that, 15.18% in *C. ventricosus*, Table S4 and Table S5), and it belonged to the LTRs of Class I retrotransposons. Given the critical roles of TEs in genome evolution and function, including genome mutations, rearrangements, and the promotion of new gene formation [14], we hypothesize that TE hotspots in crucial regions may be associated with the high diversity and hypervariability observed in conopeptides.

Furthermore, extensive research has revealed that gene flow between genetically distinct populations is a common occurrence in nature. In fact, it has been observed that introgressive hybridization between species can confer selective advantages to the receiving population, serving as a driving force behind the evolution of adaptive phenotypes [26]. The genus *Conus*, estimated to comprise approximately 700 species [1], has undergone rapid speciation through adaptive radiation, which likely promotes the occurrence of introgressive hybridization among different *Conus* lineages [27]. This notion is supported by studies documenting hybridization and introgression in various genetic regions, including mitochondrial genomes, nuclear gene regions, and conotoxin loci, within several *Virroconus* species. These findings strongly suggest that introgressive hybridization plays a significant role in the adaptive radiation of Conidae [28]. Similarly, our study provides clear evidence of introgressive hybridization involving conotoxin genes among multiple *Conus* species, indicating that introgressive hybridization is a frequent phenomenon within the genus *Conus*. This phenomenon is likely a contributing factor to the observed genetic diversity in conotoxin genes.

## Conclusions

Our study provides valuable insights into the genetic diversity and evolution of conotoxin genes in *Conus* species. We observed species-specific variations, evidence of positive selection, and higher divergence in conotoxin coding regions. Notably, our investigation uncovered transposable element hotspots in the flanking regions (both upstream and downstream) of conotoxin genes, as well as in the introns adjacent to the highly diverse mature peptide of conotoxins. It implies that these transposable element-rich regions play a crucial role in driving the extensive diversity observed in conopeptides. Additionally, our study detected robust signals of introgressive hybridization involving conotoxin genes across numerous species of *Conus*, highlighting the significant impact of introgressive hybridization on the genetic diversity of conotoxin genes and the overall evolution of the *Conus* genus. These findings contribute to our understanding of the molecular mechanisms underlying the hypervariability of conotoxin genes.

## Materials and methods

### Assembly of *Conus* transcriptomes

Transcriptome sequencing datasets of 34 *Conus* species were downloaded from NCBI (Table S1). Adapters and low quality bases of the sequencing reads were filtered using Trimmomatic-v0.36 [29] with as parameters: “LEADING:3 TRAILING:3 SLIDINGWINDOW:4:15 MINLEN:80”, and the quality of reads were checked by FastQC (https://www.bioinformatics.babraham.ac.uk/projects/fastqc/). Transcripts of each species were assembled using Trinity-v2.6.6 [30] with as parameters: “--min_kmer_cov 2 --min_glue 3 --no_normalize_reads”, followed by clustering of the predicted transcripts into unigenes by using Corset-v1.09 [31] with default parameters, respectively. The N50 of each resulting transcriptome species were calculated using a homemade Perl script and the completeness evaluation was performed using BUSCO-v5.4.7 [32] with default parameters using metazoa_odb10. TransDecoder-v5.3.0 (https://github.com/TransDecoder/) was used for prediction of ORF in unigenes, followed by functional annotation using eggNOG-mapper-v2.0.1 [33] with as parameters: “--target_orthologs all -m diamond”.

### Prediction and identification of conotoxins in *Conus* transcriptomes

Mapping of CDS sequences of 34 *Conus* species against the reference conotoxin peptides from ConoServer database [34] was performed by using Diamond-v0.9.22 [35] with as parameters: “-p 30 -k 10 -e 1e-5”. Also, the Hidden Markov Model (HMM) method was used to identify the conotoxin candidates in each species of *Conus*. A profile HMM from previous research [36] was used as reference, and prediction of conotoxins was performed by HMMER-v3.1b2 [37] with as parameters: “-E 1e-5 --domE 1e-5”. All candidate conotoxins predicted from homology and HMM search in each species of *Conus* were merged and subjected to redundancy removal. Furthermore, non-redundant candidate conotoxins were searched against the non-redundant protein sequences database (NR) using Diamond-v0.9.22, and non-conotoxins were filtered by manual curation. Finally, domains in each conotoxins were identified by ConoPrec [34].

### Calculation of Ka/Ks in conotoxins

OrthoFinder-v2.2.1 [38] with the parameters: “-S diamond -og -t 30 -a 30” was used to infer orthogroup of the predicted conotoxins from 34 *Conus* species. And combined with the corresponding CDS of each conotoxins, ParaAT-v2.0 [39] was used to calculate the Ka/Ks within each of the conotoxins orthogroup pairs with as parameters: “-p proc -m mafft -f axt -g -t -k”, followed by filtering with the threshold of *p*-value (Fisher) < 0.01. The visualization of Ka/Ks results was performed using ggplot2 [40].

### Genome reassembly of *C. betulinus*

Whole genome sequencing datasets of *C. betulinus* from recently published research [10], including Illumina and PacBio sequencing platform, were obtained from NCBI (PRJNA578609). For Illumina sequencing datasets, adapters and low quality bases of reads were filtered using Trimmomatic-v0.36 and checked with FastQC, followed by removing duplication by using Nubeam-dedup [41] with default parameters. In addition, 1 million reads in each sequencing dataset were randomly selected using seqtk (https://github.com/lh3/seqtk), and against the non-redundant nucleotide database (NT) using blastn [42] to make taxonomic assignments. A homemade Perl script was used to summarize the taxa of reads and analyze the potential contamination organisms, followed by removing contaminated reads using bbmap-v38.08 [43] with default parameters. For PacBio sequencing datasets, combined with the pretreatment of Illumina sequencing reads, FMLRC2-v0.1.3 [44] was used to perform the error correction of reads with as parameters: “-t 40 -C 10”. 50,000 corrected reads in each dataset were randomly selected by a homemade Perl script, and blastn was used against the NT database. Contaminated organisms were summarized, followed by removal using minimap2-v2.11 [45] with default parameters.

PacBio raw reads were used to perform genome assembly using wtdbg2-v2.5 [46] with as parameters: “-A -S 3 -X 50 -l 5000 -x sq -g 2.5 g -t 40”, and consensus sequences were generated and polished using wtpoa-cns. Furthermore, contigs were scaffolded using LRScaf-v1.1.11 [47], and polished using NextPolish-v1.3.1 [48] with corrected Illumina reads. Finally, sequences that were longer than 1 kb were retained, and blastn was used against the NT database and the mitochondrion genome of *C. betulinus* (MG924728.1) to make taxonomic assignments. Possible contamination, such as bacteria or mitochondrion, was manually filtered.

### Repeats and genome annotation of *C. betulinus*

*De novo* repeat library was constructed using RepeatModeler-v1.0.11 [49]. Additionally, LTR_Finder-v1.07 [50] with as parameters: “-D 20000 -d 1000 -L 7000 -l 100 -p 20 -M 0.9 -C”, and LTRharvest [51] with as parameters: “-similar 90 -vic 10 -seed 20 -seqids yes -minlenltr 100 -maxlenltr 7000 -mintsd 4 -maxtsd 6” was used to identify the LTR retrotransposons, respectively. And a high-quality LTR library was generated using LTR_retriever-v2.9.0 [52] with default parameters. Subsequently, combined with the results of RepeatModeler, LTR_retriever and Repbase database [53], transposable elements (TEs) were identified and classified by performing with RepeatMasker-v4.0.7 [54]. Furthermore, gene structures were firstly predicted using MAKER2 [55] together by *de novo*, homology and RNA-seq methods. In addition, PASA-v2.3.3 [56] and Stringtie-v1.3.4d [57] were used to optimize the predicted gene structures, respectively. Finally, gene models predicted from MAKER2, PASA and Stringtie were integrated by EVidenceModeler [58] into a comprehensive and non-redundant set of gene structures.

Conotoxin genes in *C. betulinus* were identified as described above. Additionally, gene structures, especially the coordinates of exons of conotoxin genes, were manually checked and revised using Exonerate [59]. Finally, completeness evaluation of all protein-coding genes was performed using BUSCO-v5.4.7 [32] using metazoan_odb10.

### Whole genome duplication and gene family analysis of *C. betulinus*

WGDdetector-v1.1 [60] was used to perform the whole genome duplication (WGD) analysis of *C. betulinus*. Meanwhile, conserved homologous gene blocks in *C. betulinus* was detected using MCScanX [61], and visualized using R package RIdeogram [62]. Additionally, recently published genome of *C. ventricosus* (GCA_018398815.1) [11] was also used for WGD and conserved homologous gene blocks analysis.

Orthogroups were identified among 8 selected species of gastropods, namely, *Achatina fulica* [63], *Aplysia californica* (GCF_000002075.1), *Biomphalaria glabrata* (GCA_000457375.1), *Chrysomallon squamiferum* [64], *Elysia chlorotica* (GCA_003991915.1), *Lottia gigantea* (GCF_000327385.1), *Pomacea canaliculata* (GCA_003073045.1) and *C. betulinus* using OrthoFinder-v2.2.1 [38] with the parameters of “-S diamond -M msa -t 30 -a 30 -T fasttree”. Subsequently, the absolute rates of molecular evolution and divergence times were inferred using r8s-v1.81 [65] with as parameters: “-s 961030 -p ‘Achatina_fulica,Pomacea_canaliculata’ -c ‘421’”, followed by identifying gene family expansion and contraction using CAFÉ-v4.2.1 [66] with default parameters. Finally, rapidly evolving family genes were functional annotated using Diamond-v0.9.22 [35] against with KEGG (Kyoto Encyclopedia of Genes and Genomes), NR and UniProt databases with the threshold of E-value 1e-5.

### Structure and expression of conotoxin genes in *C. betulinus*

Gene full length, exon and intron length, and exon number in conotoxin and non-conotoxin genes were summarized using a homemade Perl script. Visualization of statistical results was performed using ggplot2 [40].

Transcriptomes of *C. betulinus* with multiple specimens and tissues were obtained from NCBI (PRJNA290540) [36]. Three of the samples are from venom duct tissue of specimens with different body lengths, namely small, middle, and big; another sample is from venom duct that mixed with these tissues; and the last one is from venom bulb tissue of the middle specimen. Quality control of sequencing reads was performed as described above. Clean reads of each sample were mapped into genome of *C. betulinus* using HISAT2-v2.1.0 [67] with default parameters, respectively. Subsequently, paired read counts were quantified using featureCounts-v1.6.2 [68], and TPM (Transcripts Per Kilobase Million) method was used to normalize and calculate the expression of genes using a homemade Perl script. The TPM values of conotoxin genes were used to perform hierarchical clustering using pheatmap with z-score normalization and to compare the expression of conotoxin genes between different specimens using ggtern [69]. Meanwhile, alternative splicing between different specimens and tissues was performed using LeafCutter-v0.2 [70] and filtered with *p* < 0.05 for significantly alternative splicing sites, followed by visualization using ggsashimi [71].

### Introgression analysis of conotoxin genes in *Conus*

Targeted sequencing data sets of conotoxin genes from 32 cone snails (Conidae) that were published in previous research [7] were obtained from NCBI (PRJNA437715). Quality control of reads was performed as described above. Clean reads from each cone snail were mapped into the chromosome-level genome of *C. ventricosus* [11] using BWA-v0.7.17 [72]. SNPs in each cone snail were identified using GATK-v4.0.5.2 [73] and filtered with the following parameters: “QD < 2.0 || MQ < 40.0 || FS > 60.0 || SOR > 3.0 || MQRankSum < -12.5 || ReadPosRankSum < -8.0”. Finally, introgression between cone snails was performed using Dsuite-v0.4 [74].

### Electronic supplementary material

Below is the link to the electronic supplementary material.

Supplementary Material 1

Supplementary Material 2

Supplementary Material 3

Supplementary Material 4

Supplementary Material 5

Supplementary Material 6

Supplementary Material 7

Supplementary Material 8

Supplementary Material 9

## Data Availability

Transcriptome sequencing datasets of 34 *Conus* species are listed in Table S1 (Supplementary File 1). Whole genome sequencing datasets of *C. betulinus* used in this study are available from published research (PRJNA578609) [10], and targeted sequencing of conotoxin genes from 32 Conidae genomes used in this study are available from published research (PRJNA437715) [7]. All datasets are freely available on NCBI. The predicted protein-coding genes and conotoxins from the reassembled genome of *C. betulinus* in the present study are available in Supplementary Files 7 to 9. Homology clustering results for conotoxin genes that were identified from the reassembled and published genomes of *C. betulinus* are listed in Table S3 (Supplementary File 3). The annotation of repeat elements in the reassembled genome of *C. betulinus* are listed in Table S2 (Supplementary File 2). The content of major transposon elements in flanking regions of protein-coding genes and introns in conotoxin genes in *C. betulinus* and *C. ventricosus* was summarized in Table S4 to Table S6 (Supplementary File 4 to 6).
