# Supplementary material for: Systematic dissection of genomic features determining the vast diversity of conotoxins
Source: BMC Genomics. 2023 Oct 9;24:598. doi: 10.1186/s12864-023-09689-4 (PMC10561478; doi:10.1186/s12864-023-09689-4)
Supplement: Supplementary file 1 — Supplementary Material 1 [file 12864_2023_9689_MOESM1_ESM.docx]

**Supplemental material**

**Systematic dissection of genomic features determining the vast diversity of conotoxins**

Jian-Wei Zheng^1,2†^, Yang Lu^1†^, Yu-Feng Yang^1^, Dan Huang^1^, Da-Wei Li^1^, Xiang Wang^1^, Yang Gao^3^, Wei-Dong Yang^1^, Yuanfang Guan^4^, Hong-Ye Li^1*^

*^1^Key Laboratory of Aquatic Eutrophication and Control of Harmful Algal Blooms of Guangdong Higher Education Institute, College of Life Science and Technology, Jinan University, Guangzhou 510632, China*

*^2^College of Food Science and Engineering, Foshan University of Science and Technology, Foshan 528231, China*

*^3^Gulou Hospital, Nanjing University, Nanjing, China.*

*^4^Department of Computational Medicine and Bioinformatics, University of Michigan, Ann Arbor, MI, USA.*

***Corresponding to:** Hong-Ye Li, [thyli@jnu.edu.cn](mailto:thyli@jnu.edu.cn), 510632

^†^Jian-Wei Zheng and Yang Lu authors contributed equally to this work.

**Table S1 Transcriptome information of 34 species of *Conus* in the precent study**

| **Species** | **BioProject ID** | **Tissue type** | **Species** | **BioProject ID** | **Tissue type** | **Species** | **BioProject ID** | **Tissue type** |
| --- | --- | --- | --- | --- | --- | --- | --- | --- |
| *C. abbreviatus* | PRJNA658056 | Venom duct | *C. judaeus* | PRJNA658056 | Venom duct | *C. sp.* f AW-2021 | PRJNA658056 | Venom duct |
| *C. arenatus* | PRJNA298293 | Venom duct | *C. lenavati* | PRJNA275458 | Venom duct | *C. sponsalis* | PRJNA298293 | Venom duct |
| *C. aristophanes* | PRJNA658056 | Venom duct | *C. litteratus* | PRJNA586870 | Venom duct | *C. striatus* | PRJNA505200 | Venom gland |
| *C. bayani* | PRJNA704767 | Venom duct | *C. lividus* | PRJNA298293 | Venom duct | *C. terebra* | PRJNA505200 | Venom gland |
| *C. betulinus* | PRJNA290540 | Venom duct, Venom bulb | *C. magus* | PRJNA556342; PRJNA505200 | Venom duct, Venom gland | *C. textile* | PRJNA505200 | Venom gland |
| *C. chaldaeus* | PRJNA658056 | Osphradium | *C. maioensis* | PRJNA631880 | Venom gland | *C. tribblei* | PRJNA275371; PRJNA273524 | Venom duct |
| *C. imperialis* | PRJNA645157 | Venom duct | *C. marmoreus* | PRJNA505200; PRJNA298293 | Venom gland, Venom duct | *C. ventricosus* | PRJNA678883 | Venom gland, Foot muscle |
| *C. coronatus* | PRJNA298293 | Venom duct | *C. miliaris* | PRJNA257931 | Venom duct | *C. virgo* | PRJNA505200; PRJNA298293 | Venom gland, Venom duct |
| *C. ebraeus* | PRJNA298293 | Venom duct | *C. mordeirae* | PRJNA658056 | Venom duct | *C. regonae* | PRJNA658056 | Venom duct |
| *C. episcopatus* | PRJDB3896 | Venom duct, Radular sac, Salivary gland | *C. purpurascens* | PRJNA436049 | Liver, Proboscis, Foot, Eye | *C. consors* | PRJNA271554 | Venom duct, Venom bulb, Salivary glands, Proboscis, Oesphradium, Nervous ganglions, Mantle tissue, Foot tissue |
| *C. ermineus* | PRJNA433918 | Venom duct | *C. quercinus* | PRJNA298293 | Venom duct |  |  |  |
| *C. gloriamaris* | PRJNA385205; SRP021082 | Venom gland | *C. rattus* | PRJNA298293 | Venom duct |  |  |  |
