# Supplementary material for: Systematic dissection of genomic features determining the vast diversity of conotoxins
Source: BMC Genomics. 2023 Oct 9;24:598. doi: 10.1186/s12864-023-09689-4 (PMC10561478; doi:10.1186/s12864-023-09689-4)
Supplement: Supplementary file 2 — Supplementary Material 2 [file 12864_2023_9689_MOESM2_ESM.docx]

**Supplemental material**

**Systematic dissection of genomic features determining the vast diversity of conotoxins**

Jian-Wei Zheng^1,2†^, Yang Lu^1†^, Yu-Feng Yang^1^, Dan Huang^1^, Da-Wei Li^1^, Xiang Wang^1^, Yang Gao^3^, Wei-Dong Yang^1^, Yuanfang Guan^4^, Hong-Ye Li^1*^

*^1^Key Laboratory of Aquatic Eutrophication and Control of Harmful Algal Blooms of Guangdong Higher Education Institute, College of Life Science and Technology, Jinan University, Guangzhou 510632, China*

*^2^College of Food Science and Engineering, Foshan University of Science and Technology, Foshan 528231, China*

*^3^Gulou Hospital, Nanjing University, Nanjing, China.*

*^4^Department of Computational Medicine and Bioinformatics, University of Michigan, Ann Arbor, MI, USA.*

***Corresponding to:** Hong-Ye Li, [thyli@jnu.edu.cn](mailto:thyli@jnu.edu.cn), 510632

^†^Jian-Wei Zheng and Yang Lu authors contributed equally to this work.

**Table S2 Annotation of major repeat elements in the reassembled genome of *C. betulinus***

| Type | | Total number | Total length (bp) |
| --- | --- | --- | --- |
| SINEs | | 130,428 | 28,348,133 |
| major superfamilies | MIR | 20,146 | 6,811,498 |
|  | tRNA | 38,848 | 8,754,598 |
|  | tRNA-Meta | 15,807 | 1,183,713 |
| LINEs | | 709,753 | 259,632,282 |
| major superfamilies | I | 150,002 | 75,481,951 |
|  | I-Nimb | 16,304 | 6,778,327 |
|  | L2 | 80,516 | 30,983,087 |
|  | Penelope | 134,759 | 40,048,254 |
|  | RTE-X | 84,540 | 53,934,069 |
| LTRs | | 946,378 | 183,976,981 |
| major superfamilies | Copia | 52,596 | 15,111,987 |
|  | DIRS | 6,609 | 3,890,870 |
|  | ERV1 | 10,250 | 1,464,208 |
|  | ERVK | 9,213 | 765,682 |
|  | Gypsy | 320,451 | 101,116,856 |
| DNAs | | 871,061 | 112,569,110 |
| major superfamilies | CMC-EnSpm | 209,403 | 31,418,820 |
|  | Maverick | 13,733 | 1,864,719 |
|  | Novosib | 125,159 | 14,352,976 |
|  | TcMar-Tc1 | 161,674 | 32,963,930 |
| Small RNA | | 39,367 | 7,013,329 |
| Satellites | | 129,679 | 11,036,275 |
| Simple repeats | | 4,913,265 | 314,391,779 |
| Low complexity | | 374,775 | 25,947,087 |
| Unclassified | | 1,206,916 | 324,233,014 |
